# Staged open surgical repair of an aortic esophageal fistula in the setting of an aortic dissection

**DOI:** 10.1016/j.jvscit.2025.102051

**Published:** 2025-11-13

**Authors:** Sneha Thandra, Olivia Fuson, Victor Pretorius, Mark Onaitis, Mark Kearns, Ann Gaffey

**Affiliations:** a Division of Vascular and Endovascular Surgery, Department of Surgery, University of California at San Diego, San Diego, CA; b Division of Cardiovascular and Thoracic Surgery, Department of Surgery, University of California at San Diego, San Diego, CA

**Keywords:** Thoracic to aortic esophageal fistulas, TEVAR, Thoracic endovascular aortic repair, Type A aortic dissection, TAAD, Aortic dissection

## Abstract

Aortoesophageal fistula is a rare but fatal complication commonly arising from thoracic aortic aneurysms or secondarily from thoracic endovascular aortic repair. Management remains challenging with few reports of durable repair. We present a 52-year-old woman with prior type A aortic dissection repair who developed persistent pain requiring arch replacement and thoracic endovascular aortic repair. She presented 4 months later with sepsis and imaging revealing extraluminal gas consistent with aortoesophageal fistula. She underwent graft explantation, debridement, and esophageal resection with delayed esophagostomy. Thirty months postoperatively, she remains stable. This case highlights early recognition and aggressive staged surgical management as critical for favorable outcomes.

Aortic dissection is among the most severe aortic catastrophes, often complicated by rupture or malperfusion.^1^ Survivors develop progressive aneurysmal degeneration and false lumen expansion. Medical management is mainstay for uncomplicated dissections. However, patients with chronic postdissection descending thoracic and thoracoabdominal aortic aneurysms are considered for endovascular approaches like thoracic endovascular aortic repair (TEVAR), with or without false lumen occlusion.^2^ Unfortunately, endovascular surgery is not without risk. TEVAR risks include paraplegia, retrograde dissection, and aortoesophageal fistula (AEF) formation, albeit rare.^3^^,^^4^

AEF is a rare, potentially fatal pathology characterized by an abnormal connection between the aorta and esophagus. The reported annual incidence is about 1.5 per million, with a 42% in-hospital mortality rate, even with intervention. AEF is categorized as either primary (typically owing to an aortic aneurysm or foreign body) or secondary (generally owing to endovascular intervention complications).^5^ The formation of AEF after TEVAR was initially reported in 1998 and current incidence is approximately 1.7% to 1.9%.^4^^,^^6^ Although the exact mechanism of AEF development post TEVAR remains unknown, proposed mechanisms involve ischemia and inflammation owing to infection, erosion, and necrosis that result from stent graft rigidity and expanding pressures.^5^^,^^7^

The presentation of AEF usually involves a latent symptom-free period followed by massive arterial hematemesis resulting in death, or infection, subsequent sepsis, and death.^7^ Given its cataclysmic and potentially fatal nature, early identification is crucial. Diagnosis remains challenging, however, and there are few reported cases of successful management of secondary AEF (Table I).^4^^,^^8^, ^9^, ^10^, ^11^, ^12^, ^13^, ^14^, ^15^, ^16^, ^17^, ^18^, ^19^, ^20^, ^21^, ^22^ Despite limited knowledge on AEF treatment, favorable outcomes have been achieved with graft replacement and esophagectomy coupled with broad-spectrum antibiotic therapy.^23^ Below, we present a successful endovascular and staged open surgical repair of an aortic dissection and AEF. The patient consented to publication of their clinical information.

**Table:** Reported cases and management of secondary aortoesophageal fistula (*AEF*) post thoracic endovascular aortic repair (*TEVAR*) in the last 10 Years

Title	Author	Year	Age, years	Presentation/imaging	Operative intervention	Outcome	Length of follow-up
Surgical treatment of graft infection combined with aortoesophageal fistula after TEVAR: a retrospective single-center, single-arm study	Jin et al	2024	Mean 57.8 years (36-77 years); n = 10	Fever, abdominal/chest/back pain, hematemesis, air surrounding the prosthesis on contrast-enhanced CT	TEVAR (n = 2)	Death	n/a
					Open surgery (n = 6)	2 Survivals, 4 deaths	3 months (survival), 72 months (survival)
					TEVAR + open surgery (n = 2)	1 Survival, 1 death	3 months (death)
Surgical repair of an aortoesophageal fistula after salvage thoracic endovascular aortic repair: a case report	Uemura et al	2024	70	Increased inflammatory response Air involvement in the esophagus at the periphery of the intra-aneurysmal stent graft	Radical blood vessel prosthesis implantation and fistula closure	Survival	1 year
Aortoesophageal fistulae following TEVAR: Case report and literature review	Chaves et al	2023	62	GI bleeding and clinical signs of infection Prosthetic gas	Esophageal resection and gastrointestinal exclusion	Death	n/a
A Lethal Late Complication: Aortoesophageal Fistula after TEVAR	Karaka et al	2023	64	AEF on CTA	Open repair	Death	n/a
Aorto-esophageal fistula postthoracic endovascular repair of type B aortic dissection: an uncommon catastrophic complication	Deshpande et al	2021	79	Fever without hematemesisContinuous air column communicating with the false lumen	Feeding jejunostomy with broad spectrum antibiotics after patient refused palliative esophageal stenting	Death	n/a
Surgical treatment for secondary aortoesophageal fistula	Sugiyama et al	2020	Mean 67 (range, 41-78); n = 6	Fever, back pain, hemoptysis, hematemesis Air bubbles between the aorta and esophagus or fistula on endoscopy	TEVAR (n = 2)	Survival	Death (86 days), survival (28 days)
					Open repair (n = 2)	Survival	Death, (229 days), survival (209 days)
					Combined repair (n = 2)	Death	n/a
Massive hemorrhage from an aortoesophageal fistula caused by esophageal stent implantation A case report and literature review	Zhan and Xu	2019	79	Hemodynamic shock with massive hematemesis AEF with an ulcer-like projection on the aortic arch where the esophageal stent was placed	TEVAR	Death	n/a
Three-step surgical treatment of aortoesophageal fistula after thoracic endovascular aortic repair: A case report	Kamigaichi et al	2019	71	Fever Fistula between esophagus and aortic aneurysm	Three-step surgical approach: esophagectomy/cervical esophagostomy, aortic replacement, and reconstruction of the esophagus	Survival	24 months
Aortoesophageal Fistula: A Fatal Complication of Thoracic Endovascular Aortic Stent-Graft Placement	Rawala et al	2018	80	Hematemesis Thoracic aortic aneurysm and AEF	TEVAR -> esophageal stent	Recurrence -> death	3 months
Open Surgical Treatment of Secondary Aortoesophageal and Aortobronchial Fistula after Thoracic Endovascular Aortic Repair and Esophagocoloplasty in a Second Procedure	Sladojevic et al	2017	52	Dysphagia and chest pain followed by hematemesis and hemoptysis Endoscopic findings: lesion of the esophageal wall with chronic abscess formation and stent-graft protrusion into the cavity	Open repair	Survival	1 year
Thoracic aortic aneurysm complicated by secondary aortoesophageal fistula after thoracic endovascular aortic repair: a case report	Spitaels et al	2017	71	Dysphagia Rupture of the esophagus in connection with the aneurysm	Open repair	Survival	1 month
Secondary aorto-esophageal fistula after thoracic aortic aneurysm endovascular repair treated by covered esophageal stenting	Tao et al	2016	86	Upper GI bleed	Emergency gastroscopy and esophageal stent insertion	Survival	8 months (death)
Management of an aorto-esophageal fistula, complicating a descending thoracic aortic aneurysm endovascularly repaired	Georvasili et al	2016	64	Chest pain and high CRP levels	Open	Survival	2 years
Outcomes of a staged surgical treatment strategy for aortoesophageal fistula	Kawamoto et al	2015	Mean 70.9 (range, 52-83); n = 10	Hematemesis, fever, fatigue	Three-stage surgical treatment strategy: bridging TEVAR/debridement, aortic replacement, esophageal reconstruction	In-hospital mortality in the acute phase was 30%	Survival at 1 and 5 years were 68.6% and 42.9%
Emergency open surgery for aorto-esophageal and aorto-bronchial fistulae after thoracic endovascular aortic repair: a single-center experience	Luehr et al	2014	Mean 68.75 (range, 49-77); n = 8	Hematemesis, hemoptysis, melena, dysphagia, chest pain, fever	Open	Survival	In-hospital mortality of 25% within 40 days
Aorto-Esophageal Fistula After Thoracic Endovascular Aortic Repair: Successful Open Treatment	Dumfarth et al	2014	56	Hematemesis	Open	Survival	6 months

*CRP,* C-reactive protein; *CT,* computed tomography; *CTA,* computed tomography angiography; *GI,* gastrointestinal.

## Case report

**Fig 1:**
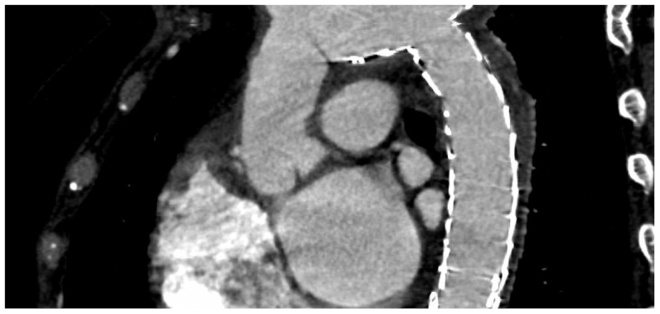
Pre-repair and fistula development computed tomography (CT) image. Centerline image of patient's chest status post ascending aorta/aortic arch graft repair, aortic debranching, and thoracic endovascular aortic repair (TEVAR) of the descending thoracic aorta for aortic dissection repair.

A 52-year-old woman with a history of type A aortic dissection status post supracoronary stent graft with residual type B dissection presented with left-sided chest pain radiating to her left shoulder and back. Computed tomography angiography (CTA) of the head and neck identified a dissection flap extending from the innominate artery to the right common carotid artery up to the right internal carotid artery. She was taken to the operating room for a redo sternotomy, zone 2 arch replacement, and complete debranching of arch vessels. Two months later, she underwent TEVAR with a Gore CTAG distal to the neosubclavian and immediately superior to the celiac artery (Fig 1). At that time, the distal visceral tear causing retrograde flow to zone 5 of the descending thoracic aorta was not felt to warrant a STABILISE technique. The superior mesenteric and left renal artery were also perfused from the false lumen. Despite the tear, the thoracic aorta showed remodeling with progressive false lumen thrombosis.

**Fig 2:**
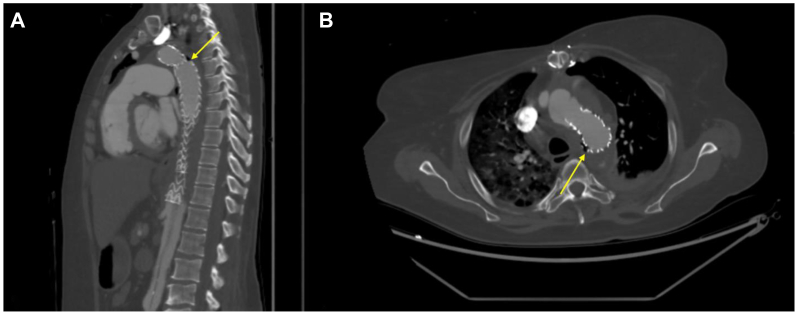
Pre-repair computed tomography (CT) scan of the chest. **(A)** Sagittal view showing extraluminal gas (*yellow arrow*) at the level of the mid-descending aorta extending toward the esophagus worrisome for aortoesophageal fistula (AEF). **(B)** Axial view of the adjacent gas (*yellow arrow*).

**Fig 3:**
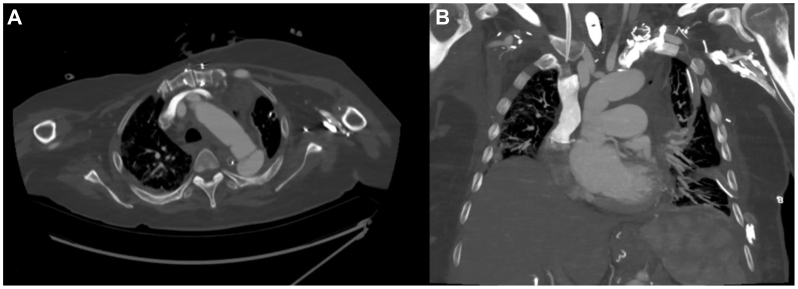
Postrepair computed tomography (CT) scan of the chest after interval resection of the descending aorta and aortic stent with graft replacement. **(A)** Axial view showing decreased gas foci adjacent to proximal descending aorta with resolution of previously seen gas along distal ascending aorta. **(B)** Coronal view.

Four months after TEVAR, she was readmitted with fevers. CTA demonstrated extraluminal gas surrounding the mid-descending thoracic aorta (zone 4) abutting the esophagus, raising concern for AEF (Fig 2). Blood cultures grew *Candida*, prompting fluconazole use and broad-spectrum antibiotics for her infected graft, with subsequent Karius testing identifying *Staphylococcus epidermidis*, for which daptomycin was added. Despite intravenous antibiotics, she deteriorated. Aspirin was held and anticoagulation was deferred. After multidisciplinary discussions, she underwent left thoracoabdominal exposure with cardiopulmonary bypass support, infected endograft explantation, aortic reconstruction with rifampin-soaked graft, and esophageal resection with a lumbar drain placed preoperatively for spinal cord ischemia prophylaxis (Fig 3). During the repair, she was maintained on left atrium to femoral bypass to ensure visceral and pelvic perfusion. Intraoperative findings confirmed bilious contamination of the mediastinum and erosion of the Gore-Tex through the aorta. Given her profound coagulopathy, the decision was made to defer esophagostomy for 24 hours. Once her coagulopathy resolved, she was discharged on aspirin and apixaban. She continued to recover and received enteral nutrition via a gastrojejunostomy tube for 11 months and then underwent gastric pull-through to reestablish gastrointestinal continuity.

Her postoperative course was complicated by hoarseness, neurogenic bladder, and lower extremity weakness. She was eventually discharged to a rehabilitation center and requires lifelong vancomycin, ceftriaxone/metronidazole, and fluconazole. Now at 30 months follow up, she has returned to work, tolerates oral intake, and has no subsequent vascular or thoracic-related admissions.

## Discussion

Although rare, secondary AEF after TEVAR is increasingly recognized with wider application of endovascular repair for aortic dissections. Mortality remains high, with sepsis and hemorrhage as leading causes.^5^ In this case, infection and persistent false lumen perfusion likely contributed to fistula development. The clinical course illustrates challenges in diagnosing and managing this condition, because the initial symptoms can be nonspecific. Imaging evidence of periaortic gas is highly suspicious and warrants urgent intervention.

Proposed mechanisms highlighted in this report include infection and inflammation. Additionally, another important problem remains: post-TEVAR residual blood flow into the false lumen, which can not only enlarge the associated aortic aneurysm, but also create susceptibility for necrosis and erosion of an already diseased aortic wall. Although the time frame for AEF formation after TEVAR is variable, studies have shown that patients who underwent TEVAR compared with graft replacement were found to have significantly shorter intervals (22.4 months vs 57.5 months, respectively).^23^ The high rate of secondary interventions and stroke risk after TEVAR remains a concern, with outcomes influenced by procedural timing and the nature of the dissection.^24^

Surgical management remains controversial, with the choice of surgical approach dependent on factors including clinical status, because not all patients are suitable for open repair. Specifically, there has been limited studies on long-term prognosis of secondary AEF after management. One systemic review found that bleeding and infection were the most common causes of death. Among patients with infected grafts, surgical treatment with a combination of aortic graft replacement and esophagectomy improved long-term survival (*P* = .043).^25^ Additionally, although previous studies have identified TEVAR as an emergency stabilization technique for AEF associated hemorrhaging, studies have found that it is associated with a five times higher odds ratio compared with open surgical repair and increases risk of AEF recurrence.^26^^,^^27^ Recently, Wang et al^28^ noted that TEVAR followed by staged surgery had a lower overall 6-month mortality rate among 56 patients compared with TEVAR alone: 20.5% vs 48.8%, respectively. The role broad-spectrum antibiotic and antifungal therapy is also notably important as an adjunct to surgical intervention, especially for patients with infected grafts and positive blood or tissue cultures in the perioperative period and for long-term prophylaxis.^25^^,^^29^

Previous reports published on secondary AEF post TEVAR, support staged surgical repair. We presented a two-stage approach targeting the infected graft before reestablishing gastrointestinal continuity. However, Fukunaga et al^30^ presented a case where they reversed these stages: esophagectomy before aortic stent graft explantation to allow patients to resume oral intake sooner. Other reports have shown that, in emergencies where infected grafts cannot be removed, simultaneous esophagectomy and vacuum therapy on exposed infected stent prosthesis followed by a staged esophageal reconstruction, have good clinical results.^31^ Although staged interventions have shown promise, limited cases of successful secondary AEF repair underscores the need for further research into long-term outcomes and optimal surgical management.

## Conclusions

Secondary AEF is a rare but devastating TEVAR complication. Early recognition with prompt surgical intervention is essential to survival. Our case supports staged open repair—including graft explantation, esophageal diversion, and long-term antimicrobial therapy—as a viable strategy. Vigilance for AEF should be maintained in patients post TEVAR with unexplained fever, periaortic gas, or gastrointestinal bleeding.

## Funding

None.

## Disclosures

None.
